# AI-Enhanced Tools and Strategies for Airborne Disease Prevention in Cultural Heritage Sites

**DOI:** 10.3390/epidemiologia5020018

**Published:** 2024-06-06

**Authors:** Enrico Greco, Anastasia Serena Gaetano, Alessia De Spirt, Sabrina Semeraro, Prisco Piscitelli, Alessandro Miani, Saverio Mecca, Stela Karaj, Rita Trombin, Rachel Hodgton, Pierluigi Barbieri

**Affiliations:** 1 Department of Chemical and Pharmaceutical Sciences, University of Trieste, Via Licio Giorgieri 1, 34127 Trieste, Italy; anastasiaserena.gaetano@phd.units.it (A.S.G.); despirt.alessia@gmail.com (A.D.S.); ssemeraro@units.it (S.S.); barbierp@units.it (P.B.); 2 Italian Society of Environmental Medicine (SIMA), Viale di Porta Vercellina, 9, 20123 Milan, Italy; prisco.piscitelli@unisalento.it (P.P.); a.miani@simaitalia.org (A.M.); 3 National Interuniversity Consortium of Material Science and Technology (INSTM), Via G. Giusti, 9, 50121 Firenze, Italy; 4 Department of Experimental Medicine, University of Salento, Via Monteroni, 73100 Lecce, Italy; 5 Department of Environmental Science and Policy, University of Milan, Via Celoria 2, 20133 Milano, Italy; 6 Department of Architecture, University of Firenze, Via della Mattonaia 14, 50121 Firenze, Italy; saverio.mecca@unifi.it; 7 Italian Academy of Biophilia (AIB), Lungadige Galtarossa 21, 37133 Verona, Italy; r.trombin@aibitalia.org; 8 Faculty of Social Sciences, European University of Tirana, Rruga Xhanfize Keko, 1000 Tirana, Albania; stela.karaj@uet.edu.al; 9 International WELL Building Institute, New York, NY 10001, USA; rachel.hodgdon@wellcertified.com

**Keywords:** Artificial Intelligence, Cultural Heritage sites, in-door Environment

## Abstract

In the wake of the COVID-19 pandemic, the surveillance and safety measures of indoor Cultural Heritage sites have become a paramount concern due to the unique challenges posed by their enclosed environments and high visitor volumes. This communication explores the integration of Artificial Intelligence (AI) in enhancing epidemiological surveillance and health safety protocols in these culturally significant spaces. AI technologies, including machine learning algorithms and Internet of Things (IoT) sensors, have shown promising potential in monitoring air quality, detecting pathogens, and managing crowd dynamics to mitigate the spread of infectious diseases. We review various applications of AI that have been employed to address both direct health risks and indirect impacts such as visitor experience and preservation practices. Additionally, this paper discusses the challenges and limitations of AI deployment, such as ethical considerations, privacy issues, and financial constraints. By harnessing AI, Cultural Heritage sites can not only improve their resilience against future pandemics but also ensure the safety and well-being of visitors and staff, thus preserving these treasured sites for future generations. This exploration into AI’s role in post-COVID surveillance at Cultural Heritage sites opens new frontiers in combining technology with traditional conservation and public health efforts, providing a blueprint for enhanced safety and operational efficiency in response to global health challenges.

## 1. Introduction

Cultural Heritage sites such as museums, historical buildings, and monuments are vital to preserving the tangible expressions of human history and culture. These sites attract millions of visitors annually, contributing significantly to education, tourism, and local economies. They are not just spaces that house historical artifacts; they are also venues where cultural narratives are interpreted and understood. The conservation of these sites is a complex task that involves maintaining their architectural integrity, protecting their artifacts, and providing a safe environment for visitors.

The COVID-19 pandemic highlighted the vulnerabilities of indoor spaces in transmitting infectious diseases [1,2]. Cultural Heritage sites, with their enclosed environments and high foot traffic, faced unique challenges [3]. The pandemic necessitated a reevaluation of health safety measures and operational protocols to mitigate the spread of the virus among visitors and staff [4,5,6]. Researchers have identified indoor air quality as a critical factor in the transmission dynamics of SARS-CoV-2, the virus responsible for COVID-19. Studies have shown that poor ventilation can significantly increase the risk of airborne transmission, especially in densely populated indoor settings [1,2,7,8], with respiratory conditions and allergic reactions being particularly relevant in the context of COVID-19. This concern is amplified in historical buildings that function as both workplaces and major tourist attractions, drawing crowds who may be unaware of the potential risks of airborne disease transmission in addition to the existing biological and biochemical hazards. The goal is to effectively mitigate these health risks, including those posed by pandemic, while preserving the historical and cultural integrity of these sites [9].

Protecting the health of individuals who frequent or work in these historical settings has become even more imperative in the face of the pandemic [10]. This includes a diverse group of individuals such as museum curators, conservation experts, researchers, and administrative staff, who face continuous exposure not only to traditional indoor environmental pollution risks but now also to the added threat of new airborne viruses [11]. The prolonged exposure to biological agents and biochemical pollutants, compounded by the risk of airborne viral transmission, underscores the need for comprehensive health protection strategies [1,2,6,12,13].

In response to these challenges, Artificial Intelligence (AI) has emerged as a potent tool for enhancing public health measures in these culturally significant spaces. AI’s application spans several dimensions, from predictive modeling and visitor behavior analysis to environmental monitoring and automated management systems. By leveraging AI, site managers can not only track and predict potential health risks but also implement more effective disease prevention strategies [4,5,12,14]. For instance, AI-driven models have been used to simulate the spread of airborne pathogens in enclosed spaces, providing valuable insights that inform ventilation improvements and crowd management.

Furthermore, AI technologies such as machine learning algorithms [15] and IoT-based sensors [16] can be integrated to monitor air quality in real-time, detect the presence of pathogens, and even control visitor flows based on real-time data. This approach not only enhances the responsiveness of health safety protocols but also ensures that these measures are based on accurate and timely information, thereby minimizing disruptions while maintaining public safety.

The integration of AI into the management of Cultural Heritage sites has led to innovative applications that address both health safety and operational efficiency. For example, smart HVAC systems [17,18,19,20] equipped with AI can optimize air filtration and circulation, reducing the risk of airborne diseases. Additionally, AI-enabled surveillance systems can analyze visitor density in real time, triggering alerts when social distancing thresholds are breached, and guiding the deployment of staff to manage crowds effectively. In the battle against airborne diseases, leveraging bioremediation stands out as an innovative and strategic approach. This method utilizes both naturally occurring and engineered systems to effectively neutralize or eliminate airborne pollutants, including pathogens. By integrating bio and photochemical remediation [21,22,23] with advanced AI-driven analytics, it is possible to enhance the detection, monitoring, and management of biological contaminants. This synergy significantly mitigates the presence of hazardous chemical and biochemical agents, as well as infectious aerosols, thereby improving air quality and reducing health risks in various environments, in particular for those artifacts standing in a very delicate equilibrium [24].

AI also plays a crucial role in the post-visit analysis by providing insights through data collected on visitor movements and interactions within the site. This data is invaluable for continuous improvement of safety measures and for planning future exhibits and events to ensure they meet health guidelines without compromising the visitor experience.

## 2. AI Application in Cultural Heritage Sites

Artificial Intelligence (AI) offers transformative potential for enhancing surveillance and operational efficiencies in Cultural Heritage sites. These applications are diverse, ranging from environmental monitoring to visitor management, each leveraging AI to mitigate health risks and enhance visitor experiences in the post-COVID era (Table 1).

One of the pivotal uses of AI in Cultural Heritage sites involves monitoring and controlling indoor air quality to prevent the spread of airborne pathogens. AI-driven systems can integrate with HVAC (Heating, Ventilation, and Air Conditioning) technologies to optimize air filtration and circulation, crucial in mitigating airborne transmission risks [17,18,19,20]. For example, machine learning algorithms can analyze data from air quality sensors to detect patterns indicating deteriorating conditions and automatically adjust HVAC operations to enhance air quality. Furthermore, AI can facilitate the deployment of advanced air purification technologies, such as HEPA filters combined with UV-C light, which have been shown to effectively neutralize pathogens like SARS-CoV-2.

AI also plays a critical role in managing visitor flows within Cultural Heritage sites [25,26] to ensure adherence to social distancing guidelines. Through the use of video analytics and IoT sensors, AI systems can monitor crowd density in real-time [27]. These systems can alert site managers when certain areas become overcrowded, enabling timely interventions to disperse crowds or close off areas temporarily. Additionally, AI can analyze visitor movement patterns to predict peak times and areas of congestion, helping in planning visitor entries and exits to minimize contact.

Predictive analytics, powered by AI, can forecast potential outbreaks by analyzing historical data and current health metrics within and around Cultural Heritage sites. By identifying potential risks before they become apparent, site managers can proactively implement preventive measures, adjust operational protocols, and communicate effectively with visitors and staff about potential health risks.

Recent advancements include the development of AI systems that can detect the presence of pathogens in the environment. These systems utilize real-time PCR (Polymerase Chain Reaction) [28] and spectroscopic analysis [29,30] to identify SARS-CoV-2-like viruses from environmental samples collected within the site. This application not only aids in early detection but also ensures that any necessary quarantine and sanitation measures can be swiftly enacted to prevent the spread of infection.

In addition to safety measures, AI can enhance the visitor experience by providing interactive guides and educational tools that respond to visitor behaviors and preferences. These systems use natural language processing and machine learning to offer personalized tours and information, enriching the cultural experience while promoting safety by reducing the need for human guides and physical touchpoints.

## 3. Challenges and Limitations

While the deployment of AI in Cultural Heritage sites presents numerous opportunities for enhancing safety and operational efficiency, it also introduces several challenges and limitations that need to be carefully addressed.

Integrating AI technologies into the existing infrastructure of Cultural Heritage sites often presents significant technical hurdles. Many of these sites are historic buildings with architectural constraints that may not easily accommodate modern technological installations. Furthermore, the accuracy and effectiveness of AI systems heavily depend on the quality and quantity of data available. In environments where data collection is limited by privacy concerns or logistical constraints, AI systems may not perform optimally.

The use of surveillance technologies and data-driven AI tools raises substantial privacy concerns [31]. Visitor tracking, facial recognition, and behavior analysis—all common in AI applications—must be managed with strict adherence to ethical standards and legal regulations regarding data protection. There is a delicate balance between enhancing safety and preserving the personal privacy of visitors, which sites must navigate carefully to avoid eroding trust and infringing on individual rights [32].

The cost of implementing and maintaining AI systems can be prohibitive for many Cultural Heritage sites, especially those that are non-profit or rely heavily on government funding. These financial constraints are compounded by the need for continuous updates and improvements to AI technologies, which require ongoing investment. Additionally, the specialized knowledge required to operate and manage these systems necessitates trained personnel, further increasing operational costs.

There is a risk of over-reliance on technology, where human intuition and expertise are undervalued. AI systems, while powerful, cannot fully replace the nuanced understanding and decision-making capabilities of human managers. Ensuring that technology complements rather than replaces human oversight is crucial for maintaining the integrity and authenticity of Cultural Heritage management.

AI solutions must be tailored to the specific needs and conditions of each site, which can vary widely in terms of size, type, visitor demographics, and environmental conditions. Scalability and adaptability are major challenges, as a one-size-fits-all approach is rarely effective. Customizing AI systems to fit the unique context of each Cultural Heritage site requires significant time and resources.

In addition to the AI-driven methodologies discussed, the integration of Internet of Things (IoT) technologies presents a significant opportunity for enhancing the monitoring and prevention of airborne infectious diseases within Cultural Heritage sites. IoT-based systems can be utilized to remotely collect, predict, and analyze data on various environmental parameters, such as humidity and temperature [33,34], which are critical for understanding the dynamics of pathogen transmission. For instance, deploying IoT sensors throughout the site can provide real-time insights into air quality and environmental conditions, enabling proactive adjustments to ventilation systems to mitigate the spread of pathogens.

IoT technologies can monitor compliance with preventive measures such as mask-wearing and hand hygiene [35]. Utilizing cameras and smart sensors, these systems can detect and alert site managers when individuals are not adhering to health protocols in risk areas, allowing for timely interventions. This approach not only enhances the overall safety of visitors and staff but also supports the enforcement of public health guidelines.

The use of IoT in conjunction with AI algorithms [15] enables a comprehensive surveillance system that can predict potential outbreaks by analyzing trends and patterns in the collected data. This predictive capability is crucial for implementing preventive measures before an outbreak occurs, thus minimizing health risks. Recent literature [36,37,38] highlights the effectiveness of IoT and AI in various public health applications, including infection control and remote monitoring, further validating the potential of these technologies in managing airborne diseases in Cultural Heritage sites.

By incorporating these solutions, Cultural Heritage sites can achieve a higher level of preparedness and responsiveness to public health threats, ensuring a safer environment for all stakeholders while preserving the integrity and accessibility of these culturally significant spaces.

## 4. Future Perspectives

The future of Artificial Intelligence (AI) in Cultural Heritage sites is poised for significant advancements that promise to further transform how these precious environments are managed and preserved. Emphasizing innovation, interdisciplinary approaches, and policy development will be key in realizing the full potential of AI in this field.

As AI technology continues to evolve, its capabilities in predictive analytics, machine learning, and real-time data processing are expected to become more sophisticated. This will enhance AI’s effectiveness in monitoring environmental conditions, managing visitor flows, and protecting artifacts. Future developments might include more advanced neural networks capable of integrating a broader range of data inputs, from environmental sensors to social media trends, to predict visitor behavior and potential health risks with greater accuracy.

The integration of AI within Cultural Heritage sites (a brief summary in Table 2) benefits greatly from an interdisciplinary approach, combining insights from conservation science, public health, data science, and visitor management. This collaborative approach not only enriches the AI applications but also ensures that they are practical, culturally sensitive, and aligned with conservation goals. Future research could explore novel ways to use AI in conjunction with traditional conservation techniques to create environments that are both safe and supportive of heritage preservation.

As AI becomes more embedded in Cultural Heritage management, there is a growing need for robust policies and regulations that address ethical concerns, data privacy, and the sustainability of AI implementations. Governments and international organizations, such as UNESCO, ICOM, ICCROM, must work together to create frameworks that facilitate the safe and effective use of AI while protecting both Cultural Heritage and individual rights [39,40]. These policies should also encourage innovation by providing guidelines that spur advancements while ensuring these technologies do not compromise the integrity of heritage sites.

The success of AI applications in Cultural Heritage will also depend on increased international collaboration and funding. Partnerships across countries can help standardize AI applications and share best practices, enhancing the global response to common challenges such as the preservation of sites and the management of public health within these spaces. Furthermore, securing adequate funding for research and the deployment of AI technologies is crucial for ensuring that heritage sites around the world can benefit from these advances.

## 5. Conclusions

The integration of Artificial Intelligence (AI) into the management of Cultural Heritage sites offers a transformative approach to enhancing public health measures and operational efficiency in these important spaces. As we have explored, AI applications can significantly improve air quality management, crowd control, predictive analytics for health safety, and pathogen detection, thereby ensuring a safer environment for visitors and staff alike. However, the deployment of such technologies is not without its challenges, including ethical concerns, technical limitations, financial constraints, and the need for tailored solutions that respect the unique characteristics of each site.

Looking ahead, the future of AI in Cultural Heritage management is bright, with promising advancements in technology, interdisciplinary approaches, and policy development poised to further enhance its effectiveness and applicability. It is essential that as we continue to harness the power of AI, we do so with a careful balance of innovation and caution, ensuring that these tools complement traditional conservation efforts and adhere to ethical standards.

Ultimately, the goal is to preserve the integrity and historical significance of Cultural Heritage sites while adopting modern solutions to address contemporary challenges. By embracing AI as a part of a broader strategy that includes human expertise and traditional practices, we can safeguard our Cultural Heritage for future generations while adapting to the evolving landscape of global health and safety standards.

## Figures and Tables

**Table 1:** AI Applications in Cultural Heritage Sites.

AI Application	Description	Limitations
Indoor Air Quality Monitoring	AI-driven systems integrate with HVAC to optimize air filtration and circulation, detect patterns indicating deteriorating air quality, and deploy advanced purification technologies.	Technical hurdles due to architectural constraints, reliance on data quality, and continuous need for updates.
Visitor Flow Management	AI systems monitor crowd density in real-time using video analytics and IoT sensors, alerting managers to overcrowded areas and predicting peak times for better planning.	Privacy concerns, need for strict adherence to ethical standards, and balancing safety with visitor privacy.
Predictive Analytics for Health Safety	AI analyzes historical data and current health metrics to forecast potential outbreaks, enabling proactive preventive measures and effective communication with visitors.	Financial constraints, especially for non-profit sites, and the need for specialized knowledge to manage AI systems.
Pathogen Detection	Real-time PCR and spectroscopic analysis detect the presence of pathogens, aiding in early detection and swift implementation of quarantine and sanitation measures.	Over-reliance on technology may undervalue human expertise and decision-making capabilities.
Interactive Guides and Educational Tools	Natural language processing and machine learning provide personalized tours and information, enhancing cultural experiences while promoting safety.	Need for tailored solutions to fit the unique context of each site, which can vary widely in terms of size, type, and visitor demographics.

**Table 2:** AI Subjects in Cultural Heritage Sites.

AI Subject	Description
Environmental Monitoring	Utilizing AI to monitor and control indoor environmental conditions, ensuring optimal air quality and pathogen detection.
Health Safety Protocols	Implementing AI-driven health safety measures to predict and prevent potential outbreaks and ensure visitor and staff safety.
Visitor Management	Using AI to manage visitor flows, monitor crowd density, and optimize entry and exit points to maintain social distancing.
Educational Enhancement	Enhancing visitor experience through AI-powered interactive guides, personalized tours, and educational tools.
Operational Efficiency	Improving overall site operations through AI applications in environmental monitoring, visitor management, and health safety protocols.

## Data Availability

The data are contained within the article.
